# Normative range of blood biochemical parameters in urban Indian school-going adolescents

**DOI:** 10.1371/journal.pone.0213255

**Published:** 2019-03-07

**Authors:** Khushdeep Bandesh, Punam Jha, Anil K. Giri, Raman K. Marwaha, INDICO, Vinod Scaria, Nikhil Tandon, Dwaipayan Bharadwaj

**Affiliations:** 1 Genomics and Molecular Medicine Unit, CSIR–Institute of Genomics and Integrative Biology, New Delhi, India; 2 Academy of Scientific and Innovative Research, CSIR–Institute of Genomics and Integrative Biology Campus, New Delhi, India; 3 Senior consultant endocrinologist and Scientific Advisor (Projects), International Life Sciences Institute-India, New Delhi, India; 4 GN Ramachandran Knowledge Center for Genome Informatics, CSIR–Institute of Genomics and Integrative Biology (CSIR–IGIB), Delhi, India; 5 Department of Endocrinology and Metabolism, All India Institute of Medical Sciences, New Delhi, India; 6 Systems Genomics Laboratory, School of Biotechnology, Jawaharlal Nehru University, New Delhi, India; University of Pisa, ITALY

## Abstract

Adolescence is the most critical phase of human growth that radically alters physiology of the body and wherein any inconsistency can lead to serious health consequences in adulthood. The timing and pace at which various developmental events occur during adolescence is highly diverse and thus results in a drastic change in blood biochemistry. Monitoring the physiological levels of various biochemical measures in ample number of individuals during adolescence can call up for an early intervention in managing metabolic diseases in adulthood. Today, only a couple of studies in different populations have investigated blood biochemistry in a small number of adolescents however, there is no comprehensive biochemical data available worldwide. In view, we performed a cross-sectional study in a sizeable group of 7,618 Indian adolescents (3,333 boys and 4,285 girls) aged between 11–17 years to inspect the distribution of values of common biochemical parameters that generally prevails during adolescence and we observed that various parameters considerably follow the reported values from different populations being 3.56–6.49mmol/L (fasting glucose), 10.60–199.48pmol/L (insulin), 0.21–3.22nmol/L (C–peptide), 3.85–6.25% (HbA_1_c), 2.49–5.54mmol/L (total cholesterol), 1.16–3.69mmol/L (LDL), 0.78–1.85mmol/L (HDL), 0.33–2.24mmol/L (triglycerides), 3.56–11.45mmol/L (urea), 130.01–440.15μmol/L (uric acid) and 22.99–74.28μmol/L (creatinine). Barring LDL and triglycerides, all parameters differed significantly between boys and girls (p< 0.001). Highest difference was seen for uric acid (p = 1.3 x10^-187^) followed by C–peptide (p = 6.6 x10^-89^). Across all ages during adolescence, glycemic and nitrogen metabolites parameters varied markedly with gender. Amongst lipid parameters, only HDL levels were found to be significantly associated with gender following puberty (p< 0.001). All parameters except urea, differed considerably in obese and lean adolescents (p< 0.0001). The present study asserts that age, sex and BMI are the essential contributors to variability in blood biochemistry during adolescence. Our composite data on common blood biochemical measures will benefit future endeavors to define reference intervals in adolescents especially when the global availability is scarce.

## Introduction

Blood tests are routinely prescribed in healthcare systems to analyze the biochemistry to determine a diseased or a healthy state. A panel of blood tests commonly measures metabolic parameters–glycemic (glucose, insulin), lipids (total cholesterol, LDL, HDL, triglycerides), nitrogen metabolites (urea, creatinine, uric acid); important electrolytes (Na^+^, K^+^, Cl^-^, HCO_3_^-^) and crucial enzymes (liver aminotransferases and phosphatases, etc.) [1]. These biochemical parameters vary considerably depending upon an individual's age, sex, ethnicity, dietary intake or physiological state of the body [2–8].

Age is a major factor that contributes to variation in blood biochemistry [9]. Ageing is a continuous dynamic process that leads to physiological, psychological and behavioral changes resulting in biological aberrations, metabolic dysfunction, cellular senescence or decline in function of organ systems [10, 11]. Amongst various life stages–infancy, childhood, adolescence, adulthood and old age, adolescence is the critical one. It marks the period of transition from childhood to adulthood and is characterized by rapid biological, social and emotional changes [12] that severely impacts future health. This transition is largely governed by gender of an individual that gives rise to difference in hormonal levels among boys and girls at puberty [13]. On account of these multiple significant physiological changes, biochemical parameters tend to vary drastically. Administering the variability of these parameters during adolescence can layoff allied metabolic diseases in adulthood. Currently, a large body of studies have examined variability in blood biochemistry in children in different populations, however only a few inspected adolescents. In view, we analyzed physiological levels of common biochemical parameters in blood (glucose, insulin, C–peptide, HbA_1_c, total cholesterol {TC}, LDL, HDL, triglycerides, urea, creatinine and uric acid) and studied their variation with Body Mass Index (BMI) in several adolescents (N = 7,618), a number that has never been studied previously. The samples were collected as a part of another ongoing project beyond the intentions to define reference intervals. Therefore, we conveniently aimed to look at the mere dispersion of values of different parameters that normally exists during adolescence.

The physiological changes during adolescence always occur in a universal order however, their timing and pace vary depending upon environmental influences (e.g. food habits, lifestyle, and cultural systems) [14–17]. Today, several populations worldwide have encountered rapid urbanization swiftly changing the lifestyle of its people and hence predisposing them to several complex diseases. These changes together with the genetic makeup determine population specific normal range of values for common biochemical parameters. One such population that exhibits huge diversity on grounds of both genetic and lifestyle ways is Indians. Indians amounting to one-sixth of world's total population [18], comprise several endogamous groups that are genetically distinct and highly diverse in terms of dietary intake and ways of living [19]. Indian diets are typically carbohydrate rich compared to the dietary patterns favored in rest of the world [20, 21], and indeed pose Indians at a high risk for metabolic diseases [21–24]. Therefore, we exclusively studied Indians at a younger age to institute an early intervention and management of highly prevalent metabolic diseases in the adult population.

## Materials and methods

The study was carried out in accordance with the principles of the Helsinki Declaration and approved by Human Ethics Committee of All India Institute of Medical Sciences, New Delhi, India and CSIR–Institute of Genomics and Integrative Biology, New Delhi, India. A written informed consent was obtained from parents/guardians of each participant followed by a verbal consent from participants themselves.

### Study population

We conducted the study at CSIR–Institute of Genomics and Integrative Biology, New Delhi, India. A total of 7,618 adolescents (3,333 boys and 4,285 girls) aged between 11–17 years were enrolled from various schools located in urban areas of Delhi–National Capital Region as a part of a study on childhood obesity conducted previously in our laboratory [25]. Average age in total sample group was observed to 13.73 years (± 1.80 years), 13.68 ±1.78 years in boys and 13.77 ±1.77 years in girls. Residing in north part of Indian subcontinent, all participants comprised Indo–European ethnicity and were selected through a pre–designed questionnaire assessing their age, dietary habits, state of birth, parent's state of origin and, past and present medical history. Participants who suffered from any chronic ailments, under medication, history of hospitalization in last six months and self–denial for blood withdrawal were excluded from the study.

The samples were categorized as Obese (OB), Overweight (OW) and Lean or Normal weight (NW) by calculating BMI z-scores and inspecting the previously established BMI percentiles for specific age and sex in children during adolescence (CDC Growth Charts) [26, 27]. BMI at or above 95^th^ percentile at same age and sex was counted as Obese [26]. BMI below 95^th^ percentile but at or above 85^th^ percentile was considered Overweight and BMI in-between 85^th^ percentile and 5^th^ percentile was defined as Lean [26].

### Sample collection and biochemical analysis

Participants were subjected to an overnight fast and their peripheral blood was collected by trained phlebotomists in three different vacutainers–serum separator tubes, sodium fluoride vacuette and potassium EDTA tubes manufactured by Becton Dickinson, New Jersey, USA. Samples were transported to laboratory over ice for processing within 2 hours of collection. Different biochemical parameters were measured using well-recognized robust analyzers–COBAS Integra 400 plus and COBAS e411. Details regarding assay methods for studied clinical parameters have been described previously [28].

### Statistical analyses

As mentioned, we wanted to capture the natural spread of values for each parameter during adolescence, so no outliers were deleted. Thus, instead of presenting the mean values that are normally affected by the outliers, we rather looked at the median values and interquartile ranges (2.5 percentile and 97.5 percentile). Mann–Whitney *U* test was used for comparing difference among different groups that were analyzed. P value less than 0.05 was considered significant. R version 3.4.0 was used for statistical analysis (http://www.R-project.org/). Although there were a small number of tests (11 parameters) that were performed between different groups, the observed differences were further reviewed after applying Bonferroni correction wherein the corrected p value was assumed to be 0.0045 (α/number of comparisons i.e. 0.05/{11}).

### Power calculation

The power of the study was calculated using two different tools separately–OpenEpi, an open-source tool for computing epidemiologic statistics for public health [29] and G*Power 3, a robust power analysis program for the social, behavioral, and biomedical sciences [30]. Power of a two-sided *U* test of each parameter was computed at α level of 0.05 and 95% confidence intervals from the respective effect size in boys and girls (i.e. = {difference in group means}/{standard deviation}) (S1 Table).

## Results

For nearly all studied biochemical parameters, the power of the study was observed to be 100 percent (97 percent for TC) to detect genuine differences between various groups (S1 Table). For LDL and TG, overall comparison among all adolescent boys and girls lacked sufficient power, however their individual comparisons at specific age groups secured necessary power.

### Glycemic parameters are strongly associated with gender and adolescence age

Normative range of plasma glycemic parameters in Indian adolescents was 3.56–6.49mmol/l (fasting glucose), 10.60–199.48pmol/l (insulin), 0.21–3.22nmol/l (C–peptide) and 3.85–6.25% (HbA_1_c) (Table 1).

**Table 1:** Normative range of common biochemical parameters in Indian adolescents.

Age group (years)	11–17 (Total)			11–17 (Boys)			11–17 (Girls)			P value
Parameters	N	Median (Percentiles 2.5–97.5)	Mean	N	Median (Percentiles 2.5–97.5)	Mean	N	Median (Percentiles 2.5–97.5)	Mean	
**FPG (mmol/L)**	6977	4.79 (3.56–6.49)	4.80	3087	4.88 (3.61–6.88)	4.91	3890	4.70 (3.52–6.16)	4.72	< 0.0001
**Insulin (pmol/L)**	5536	57.45 (10.60–199.48)	68.90	2480	52.12 (10.50–199.12)	65.34	3056	61.12 (10.75–198.84)	71.79	< 0.0001
**C-peptide (nmol/L)**	5084	1.09 (0.21–3.22)	1.26	2189	0.79 (0.19–2.98)	1.03	2895	1.35 (0.25–3.33)	1.43	< 0.0001
**HbA**_**1**_**c (%)**	3725	5.16 (3.85–6.25)	5.16	1795	5.09 (3.86–6.17)	5.08	1930	5.28 (3.86–6.34)	5.24	< 0.0001
**TC** **(mmol/L)**	6981	3.70 (2.49–5.54)	3.78	3089	3.67 (2.47–5.41)	3.74	3892	3.74 (2.51–5.62)	3.81	0.0001
**LDL (mmol/L)**	7000	2.15 (1.16–3.69)	2.20	3100	2.14 (1.15–3.63)	2.19	3900	2.16 (1.17–3.72)	2.22	0.10
**HDL (mmol/L)**	7002	1.19 (0.78–1.85)	1.22	3099	1.17 (0.78–1.81)	1.20	3903	1.20 (0.78–1.88)	1.24	< 0.0001
**TG (mmol/L)**	6982	0.96 (0.33–2.24)	1.04	3086	0.95 (0.36–2.30)	1.04	3896	0.96 (0.33–2.16)	1.03	0.81
**Urea (mmol/L)**	4860	6.75 (3.56–11.45)	6.94	2302	7.18 (3.91–11.63)	7.32	2558	6.39 (3.35–11.21)	6.60	< 0.0001
**Uric acid (μmol/L)**	5064	257.55 (130.01–440.15)	268.51	2419	297.99 (149.83–470.55)	300.27	2645	231.97 (120.74–364.55)	239.45	< 0.0001
**Creatinine (μmol/L)**	4830	42.44 (22.99–74.28)	44.02	2296	44.21 (25.64–78.36)	46.29	2534	40.67 (22.11–69.85)	41.96	< 0.0001

Values for biochemical parameters have been presented as Median (2.5 percentile and 97.5 percentile). N: sample number; FPG: fasting plasma glucose, HbA_1_c: glycosylated hemoglobin, TC: total cholesterol, LDL: low–density lipoprotein cholesterol, HDL: high–density lipoprotein cholesterol, TG: triglycerides. Mann Whitney *U* test was used to compare difference for biochemical parameters between boys and girls.

#### Plasma glucose

Boys consistently showed significantly higher levels of fasting plasma glucose than girls across all ages during adolescence (p ≤ 0.001) (Table 2, S2 Table). Mean value of plasma glucose was observed to be 4.91mmol/L in boys and 4.72mmol/L in girls (p = 6.2 x 10^−31^) (Table 1). Between subsequent ages, both boys and girls show consistent plasma glucose levels, except for age of 14 years, where plasma glucose levels in boys were observed to decline significantly than other ages during adolescence (Table 2).

**Table 2:** Variation of glycemic parameters with gender across different ages during adolescence.

Glycemic parameters	Fasting plasma glucose (mmol/L)		Fasting insulin (pmol/l)		C-peptide (nmol/l)		HbA_1_c (%)	
	Boys	Girls	Boys	Girls	Boys	Girls	Boys	Girls
**11 years**	4.81 (3.67–5.75)	4.65 (3.58–5.74)***	50.21 (11.70–170.96)	61.39 (12.61–224.46)***	0.64 (0.20–2.71)	1.15 (0.25–3.14)***	4.94 (4.0–6.01)	5.19 (4.05–6.22)***
**N**	382	473	309	345	293	331	238	208
**12 years**	4.83 (3.82–5.94)	4.69 (3.60–5.82)***	50.14 (9.44–189.55)	65.53 (13.36–215.78)***	0.87 (0.22–2.97) ^**$ $ $**^	1.38 (0.21–4.12)***^,^^**$ $ $**^	5.02 (3.86–6.22) ^**$**^	5.49 (4.16–6.40)***^,**$ $**^
**N**	523	635	421	498	387	483	283	314
**13 years**	4.90 (3.69–6.25)	4.76 (3.58–6.10)***	49.57 (11.06–158.91)	59.48 (10.18–191.72)***^,^^**$**^	0.74 (0.21–3.09)	1.37 (0.28–3.25)***	5.10 (3.59–6.23)	5.34 (3.39–6.44)***
**N**	590	733	485	607	428	575	360	369
**14 years**	4.83 (3.44–6.82) ^**$**^	4.73 (3.51–6.62)**	52.43 (10.14–181.40)	62.64 (9.47–199.5)***	0.70 (0.20–3.05)	1.41 (0.33–3.14)***	5.11 (3.85–6.13)	5.20 (4.0–6.19) ^**$ $**^
**N**	579	684	483	523	404	491	310	331
**15 years**	4.87 (3.49–7.21)	4.6 (3.38–6.70)***	53.34 (12.26–209.78)	61.60 (9.69–186)	0.82 (0.17–3.20)	1.31 (0.30–3.50)***	5.14 (4.22–6.22)	5.27 (4.00–6.31)*
**N**	430	564	346	453	285	426	260	287
**16 years**	4.85 (3.56–7.35)	4.70 (3.41–7.33)***	60.14 (10.17–203.13)	61.05 (9.00–188.42)	0.82 (0.11–2.75)	1.32 (0.26–3.03)***	5.17 (4.11–5.90)	5.21 (3.94–6.17)*
**N**	359	481	279	386	251	361	217	244
**17 years**	4.78 (3.62–7.24)	4.62 (3.51–6.26)**	53.62 (7.53–245.92)	53.98 (11.13–200.54) ^**$ $**^	1.04 (0.22–3.55) ^**$ $**^	1.43 (0.27–2.94)*	5.16 (4.20–6.20)	5.15 (3.4–6.17)
**N**	224	320	157	244	141	228	127	177

Values for biochemical parameters have been presented as Median (2.5 percentile and 97.5 percentile). N: sample number. Mann Whitney *U* test was used to compare difference in glycemic parameters between boys and girls of same age group and also for calculating difference between different age groups of boys and girls separately.

*p ≤ 0.01

**p ≤ 0.001

***p ≤ 10^−4^ for comparisons between boys and girls.

^**$**^p ≤ 0.05

^**$ $**^p ≤ 0.01

^**$ $ $**^p ≤ 0.001 for comparison within same sex with the adjacent younger age group.

#### Plasma insulin

Fasting plasma insulin levels were remarkably higher in girls than boys (p = 5.1 x 10^−15^) (Table 1). This observation prevailed over the ages from 11 years to 14 years (p ≤ 10^−4^), but at later ages of adolescence both boys and girls featured comparable plasma insulin levels (Table 2). Highest insulin levels were observed to be 65.53pmol/L (median value) at age of 12 years in girls and 60.14pmol/L in boys at age of 16 years (Table 2). Among boys, we noticed that plasma insulin levels change negligibly with age (Table 2, S3 Table). However, in girls, plasma insulin levels change significantly between ages of 12 to 13 years (p = 0.01) and 16 to 17 years (p = 0.008) (Table 2, S4 Table).

#### C–peptide

Alike plasma insulin, significantly higher levels of C–peptide were found in girls than boys across different ages in adolescence (p ≤ 10^−7^{11–16 years}, p = 0.046 {16–17 years}) (Table 2). Mean C–peptide levels were 1.43nmol/L in girls in contrast to 1.03nmol/L in boys (p = 6.6 x 10^−89^) (Table 1). Furthermore, C–peptide levels varied significantly between ages of 11–12 years in both genders–boys (p = 4.73 x 10^−4^) and girls (p = 1.88 x 10^−4^) (S3 and S4 Tables). Additionally, boys showed markedly different C–peptide levels between 16 and 17 years of age (p = 0.002) (Table 2).

#### HbA_1_c

Mean levels of HbA_1_c were noted to be 5.24% in girls and 5.08% in boys (p = 3.0 x 10^−17^) (Table 1). With increasing age in adolescence, girls showed unusually higher HbA_1_c levels than boys (p < 10^−5^ {11–13 years}, p = 0.01{15–16 years}) (Table 1). Both boys and girls exhibited significantly different HbA_1_c levels between ages of 11 and 12 years (p = 0.02 and 0.003) (S3 and S4 Tables). Additionally, girls aged 14 years displayed drastically lower HbA_1_c levels than their younger mates (p = 0.003) (Table 2).

### Amongst blood lipids only HDL varies robustly with gender and age throughout adolescence

In Indian adolescents, plasma levels for different lipids were commonly seen in the range of 2.49–5.54mmol/L (TC), 1.16–3.69mmol/L (LDL), 0.78–1.85mmol/L (HDL) and 0.33–2.24mmol/L (triglycerides) (Table 1). Indian girls had significantly higher TC and HDL levels than boys (respective p = 1.3 x 10^−4^ and 5.3 x 10^−11^) (Table 1).

#### Total Cholesterol

Across all ages during adolescence, boys and girls revealed identical plasma TC levels except at age of 14 years, when girls have robustly higher levels than boys (p = 2.44 x 10^−4^) (Table 3). Barring the age of 14 years in boys, no significant change in levels of plasma TC was observed with increasing age during adolescence within each gender (S3 and S4 Tables).

**Table 3:** Changes in lipid profile in adolescent boys and girls with age.

Lipid parameters	Total cholesterol (mmol/L)		LDL-cholesterol (mmol/L)		HDL-cholesterol (mmol/L)		Triglycerides (mmol/L)	
	Boys	Girls	Boys	Girls	Boys	Girls	Boys	Girls
**11 years**	3.78 (2.48–5.92)	3.74 (2.51–5.40)	2.20 (1.17–3.80)	2.15 (1.24–3.54)	1.19 (0.81–1.78)	1.19 (0.73–1.85)	0.95 (0.34–2.19)	1.05 (0.34–2.36)***
**N**	386	475	386	476	386	477	377	475
**12 years**	3.70 (2.51–5.31)	3.73 (2.58–5.57)	2.15 (1.10–3.62)	2.12 (1.16–3.65)	1.19 (0.76–1.92)	1.19 (0.80–1.88)	0.91 (0.30–2.22)	0.98 (0.30–2.29)*^,^^**$**^
**N**	530	644	532	643	532	645	527	641
**13 years**	3.67 (2.46–5.23)	3.72 (2.50–5.60)	2.12 (1.15–3.59)	2.12 (1.16–3.56)	1.17 (0.78–1.89)	1.19 (0.78–1.85)	0.94 (0.35–2.19)	0.96 (0.32–2.20)
**N**	586	731	588	731	588	731	587	729
**14 years**	3.58 (2.47–5.31) ^**$**^	3.70 (2.55–5.39)***	2.12 (1.21–3.40)	2.13 (1.23–3.68)	1.16 (0.77–1.75)	1.21 (0.78–1.86)***	0.94 (0.40–2.22)	0.96 (0.34–2.05)
**N**	577	689	580	689	580	689	580	689
**15 years**	3.64 (2.37–5.36)	3.76 (2.47–5.44)*	2.14 (1.10–3.54)	2.2 (1.10–3.67)	1.15 (0.78–1.70)	1.22 (0.79–1.80)***	0.97 (0.44–2.35) ^**$**^	0.92 (0.30–2.11)**
**N**	427	559	428	565	429	565	430	565
**16 years**	3.69 (2.62–5.44)	3.78 (2.49–5.93)	2.18 (1.22–3.84)	2.19 (1.15–4.23)	1.14 (0.78–1.72)	1.22 (0.79–1.94)***	1.03 (0.41–2.39)	0.95 (0.37–2.12)**
**N**	360	475	362	477	362	478	361	478
**17 years**	3.71 (2.46–5.85)	3.76 (2.59–5.78)	2.18 (1.22–4.15)	2.23 (1.22–3.73)	1.14 (0.75–1.74)	1.24 (0.86–1.97)***	0.97 (0.32–2.67)	0.90 (0.32–1.98)**
**N**	223	319	224	319	222	318	224	319

Values for biochemical parameters have been presented as Median (2.5 percentile and 97.5 percentile). N: sample number. Mann Whitney *U* test was used to compare difference in glycemic parameters between boys and girls of same age group and also for calculating difference between different age groups of boys and girls separately.

*p ≤ 0.01

**p ≤ 0.001

***p ≤ 10^−4^ for comparisons between boys and girls.

^**$**^p ≤ 0.05 for comparison within same sex with the adjacent younger age group.

#### LDL

Like TC, LDL levels were similar in boys and girls across different adolescence ages (Table 3). Besides, no difference was seen with increasing age in either gender (Table 3).

#### HDL

Indian boys and girls portrayed significantly distinct HDL levels from ages 14 years to 17 years (p < 0.001) (Table 3), with girls having higher HDL levels than boys. HDL levels were found to be constant over adolescence period in both the genders (Table 3, S3 and S4 Tables).

#### Triglycerides

Girls showed robustly higher triglyceride levels than boys at ages 11 and 12 years (p = 8.5 x 10^−4^ and p = 0.01), but unexpectedly lower triglyceride levels at ages 15, 16 and 17 years (p < 0.01) (Table 3, S2 Table). Within boys, triglyceride levels varied significantly between 14 and 15 years (Table 3, S3 and S4 Tables), conversely at 11–12 years among girls (Table 3, S3 and S4 Tables).

### Nitrogen metabolites show marked difference among adolescent boys and girls

Plasma nitrogen metabolites were observed to be normally in the ranges of 3.56–11.45mmol/L (urea), 130.01–440.15μmol/L (uric acid) and 22.99–74.28μmol/L (creatinine) in Indian adolescents (Table 1). Boys retained strikingly higher levels of all studied nitrogen metabolites than girls (p = 1.0 x 10^−39^ {urea}; 1.3 x 10^−187^ {uric acid} and 1.97 x 10^−28^ {creatinine}).

#### Urea

Throughout adolescence (11–17 years), girls showed dramatically low levels of plasma urea than boys (p <10^−4^) (Table 4). Across teenage years, plasma urea levels differed suggestively from ages 11–12 years and 15–16 years in boys (respective p = 0.04, 0.006), however only between ages 15–16 years in girls (p = 0.007) (S3 and S4 Tables).

**Table 4:** Status of nitrogen metabolites in adolescent boys and girls with age.

Nitrogen metabolites	Urea (mmol/L)		Uric acid (μmol/L)		Creatinine (μmol/L)	
	Boys	Girls	Boys	Girls	Boys	Girls
**11 years**	7.23 (4.27–11.44)	5.93 (3.53–10.43)***	238.22 (130.26–368.72)	226.92 (115.93–335.99)**	38.02 (23.65–68.53)	36.25 (22.75–57.32)
**N**	312	312	312	314	311	310
**12 years**	7.03 (3.69–11.42) ^**$**^	6.07 (3.37–10.81)***	255.17 (139.20–428.64) ^**$ $ $**^	239.11 (124.52–364.20)***^,^ ^**$ $**^	39.35 (22.13–68.04) ^**$**^	38.90 (20.34–66.05) ^**$ $**^
**N**	464	412	370	415	362	407
**13 years**	6.96 (4.17–11.21)	6.39 (3.32–10.83)***	281.64 (148.45–452.27) ^**$ $ $**^	234.95 (120.24–370.29)***	40.67 (24.14–67.20) ^**$**^	39.79 (20.34–66.98) ^**$**^
**N**	455	475	464	487	453	471
**14 years**	7.03 (3.51–11.66)	6.50 (3.54–10.86)***	310.49 (175.17–470.49) ^**$ $ $**^	233.76 (124.76–358.87)***	45.09 (26.53–79.16) ^**$ $ $**^	41.56 (23.87–68.97)***^,^^**$**^
**N**	421	451	461	472	420	446
**15 years**	7.17 (3.93–12.21)	6.32 (2.95–11.78)***	342.90 (204.45–506.99) ^**$ $ $**^	229.30 (126.05–373.34)***	50.40 (29.18–80.46) ^**$ $ $**^	43.33 (25.64–74.18)***^,^^**$ $**^
**N**	321	393	350	414	321	389
**16 years**	7.68 (4.11–11.82)^**$ $**^	6.78 (3.59–11.56)***^,^^**$ $**^	334.58 (218.74–500.39)	231.97 (131.15–352.72)***	54.82 (33.14–79.58) ^**$ $ $**^	45.09 (23.59–69.85)***
**N**	260	310	280	331	260	308
**17 years**	7.60 (4.53–11.44)	6.60 (3.41–11.31)***	332.20 (208.08–465.42)	222.46 (113.99–368.15)***	55.70 (39.08–81.17)	44.21 (23.03–68.08)***
**N**	169	205	182	212	169	203

Values for biochemical parameters have been presented as Median (2.5 percentile and 97.5 percentile). N: sample number. Mann Whitney *U* test was used to compare difference in glycemic parameters between boys and girls of same age group and also for calculating difference between different age groups of boys and girls separately.

*p ≤ 0.01

**p ≤ 0.001

***p ≤ 10^−4^ for comparisons between boys and girls.

^**$**^p ≤ 0.05

^**$ $**^p ≤ 0.01

^**$ $ $**^p ≤ 0.001 for comparison within same sex with the adjacent younger age group.

#### Uric acid

Plasma levels of uric acid were also remarkably low in girls than boys across all ages during adolescence (p < 0.005, 11 years and p < 10^−4^, 12 to 17 years) (Table 4). With each successive year in age from 11 years to 15 years, plasma uric acid levels increased in boys (p ≤ 10^−4^). On contrary, uric acid levels were seen to rise significantly only during transition from 11–12 years in girls (p = 0.005) (S3 and S4 Tables).

#### Creatinine

Together with urea and uric acid, Indian girls had apparently low levels of plasma creatinine than boys, with significant difference especially during the late adolescence period than boys (p < 10^−4^) (Table 1). In boys, creatinine levels were consistently found to be increased from age of 12 years to 16 years, with highest increment between 13 to 15 years (p < 10^−6^) (Table 4, S3 Table). Girls did not show a remarkable upsurge in creatinine levels during adolescence wherein levels increased only marginally from ages 11 to 15 years (p < 0.05).

### BMI status significantly governs blood biochemistry during adolescence

We categorized our samples as per their BMI z-scores and observed that levels of all biochemical parameters except urea alter drastically among OB, OW and NW adolescents (Table 5). Similar trend of association was also noted for all parameters in the respective BMI categories within each gender (Table S5a and S5b) barring FG, which however showed consistency when investigated individually in boys and girls.

**Table 5:** Variation of biochemical parameters with BMI during adolescence.

Parameters	Obese (OB)			Overweight (OW)			Normal weight (NW)			P value		
	N	Median	Mean	N	Median	Mean	N	Median	Mean	OB vs NW	OW vs NW	OB vs OW
FPG (mmol/L)	645	4.805 (3.69–6.04)	4.808	913	4.698 (3.52–6.05)	4.716	5413	4.810 (3.56–6.67)	4.818	0.956	0.0004	0.0104
Insulin (pmol/L)	548	97.890 (10.78–294.76)	112.490	755	78.660 (7.56–222.99)	87.912	4232	52.460 (12.04–149.3)	59.865	< 0.0001	< 0.0001	< 0.0001
C-peptide (nmol/L)	495	1.690 (0.29–4.49)	1.847	707	1.390 (0.28–3.59)	1.542	3881	0.983 (0.21–2.85)	1.130	< 0.0001	< 0.0001	< 0.0001
HbA1c (%)	331	5.540 (4.35–6.58)	5.558	533	5.380 (4.18–6.37)	5.356	2855	5.090 (3.77–6.16)	5.079	< 0.0001	< 0.0001	< 0.0001
TC (mmol/L)	649	4.061 (2.49–6.52)	4.197	925	3.859 (2.65–5.79)	3.953	5402	3.652 (2.45–5.28)	3.701	< 0.0001	< 0.0001	< 0.0001
LDL (mmol/L)	654	2.394 (1.39–4.45)	2.554	929	2.305 (1.26–3.96)	2.377	5411	2.108 (1.22–3.40)	2.133	< 0.0001	< 0.0001	0.0002
HDL (mmol/L)	652	1.070 (0.73–1.58)	1.096	927	1.160 (0.73–1.69)	1.169	5417	1.199 (0.78–1.89)	1.247	< 0.0001	< 0.0001	< 0.0001
TG (mmol/L)	477	1.062 (0.39–2.11)	1.162	718	0.975 (0.35–1.93)	1.009	5396	0.927 (0.33–1.98)	0.987	< 0.0001	0.0498	< 0.0001
Urea (mmol/L)	382	6.372 (4.0–10.64)	6.691	382	6.605 (4.11–10.07)	6.876	2390	6.533 (4.18–10.21)	6.654	0.774	0.534	0.489
Uric acid (μmol/L)	381	291.452 (179.04–439.56)	295.636	643	271.824 (154.08–414.49)	279.184	3974	249.816 (123.91–427.66)	258.703	< 0.0001	< 0.0001	0.0002
Creatinine (μmol/L)	377	41.557 (25.64–67.38)	42.871	645	43.326 (25.73–73.39)	45.396	3711	41.557 (23.87–69.85)	43.152	0.901	0.0001	0.0059

BMI status of samples was determined by calculating BMI z-scores and BMI-for-age and sex percentiles using standard CDC Growth charts. Values for biochemical parameters have been presented as Median (2.5 percentile and 97.5 percentile) and Mean. OB: Obese; OW: Overweight; NW: Normal Weight (Lean); N: sample number. FPG: fasting plasma glucose, HbA_1_c: glycosylated hemoglobin, TC: total cholesterol, LDL: low–density lipoprotein cholesterol, HDL: high–density lipoprotein cholesterol, TG: triglycerides. Mann Whitney *U* test was used to calculate p values.

## Discussion

Rapid growth and development during childhood administer normal circulating levels of various biochemical markers. Abnormal levels of such markers are classical indications that can potentially signal an underlying pediatric disease or early signs of an adult onset disease. These levels are strongly influenced by their rate of growth, organ maturity, hormone responsiveness, nutrition and metabolism [31, 32]. Additionally, children are immunologically naive and respond differently to infections, therefore adult normative values of various biochemical markers are not applicable in a pediatric setting. Given this background, we aimed to establish normative range of commonly studied blood biochemical parameters in Indian adolescents.

This is the first comprehensive study in 7,618 Indo–Europeans and a largest one worldwide ever conducted to examine blood biochemistry and its variation with gender and age during adolescence. Previously, Canadian Laboratory Initiative on Pediatric Reference Intervals (CALIPER) compiled health information from nearly 9000 community children of all ages from birth to 18 years but comprised only a handful of adolescents [33]. Likewise, the BCAMS Study in Chinese population inspected 3,223 schoolchildren aged 6–18 years [34]. Earlier studies investigating blood biochemistry specifically in adolescents were conducted in limited samples—Nigerians (N = 628) [35], Croatians (N = 998) [36] and Czech (N = 1,518) [37], which is much narrow than the present study. In India, a previous study examined a few surrogate markers for insulin resistance in 695 adolescents [38] and serum lipid profile in 4,245 children and adolescents aging from 6–17 years [39] but failed to deliver normative ranges for all common blood biochemical parameters assessed in a large sample set that could represent Indian population.

In accordance with Clinical and Laboratory Standards Institute (CLSI) guidelines [40], we studied various parameters in an interval between 0.025 and 0.975 fractiles of the distribution of results wherein 95% of the values resided. In adolescents, normative range for fasting plasma glucose of 3.56–6.49mmol/L was observed in Indians as against 3.77–8.27mmol/L in Nigerians [35] and 3.9–5.9mmol/L in Croatians [36]. For plasma insulin, Indian adolescents have normal levels within a range of 10.60–199.48pmol/L that is typically less than reported by a recent multiethnic study in Canada–CALIPER (15 – 345pmol/L) [33]. Mean plasma glucose and insulin levels were found to be 4.8mmol/L and 68.90pmol/L in the present study in lieu of previously reported levels of 5.19mmol/L and 87.5pmol/L in mere 695 Indian adolescents [38]. Besides, Indian boys featured considerably higher plasma glucose levels but lower plasma insulin than girls. This agrees with previous studies in normal weight adolescents from Czech [37] and Chinese populations [34] but confronts CALIPER data reporting similar insulin levels in both genders [33]. Girls are more insulin resistant than boys [41]. Adolescence period is largely predominated by puberty that is associated with a marked decrease in insulin sensitivity [42] that in turn is accompanied by compensatory increase in insulin secretion eventually leading to insulin resistance [43]. This provides a biological rationale for presence of remarkably high insulin and correspondingly low glucose levels in girls during adolescence.

Normative levels of C–peptide (0.21–3.22nmol/L) in Indians encompassed larger confines than reported levels in Canadians (0.26–2.24nmol/L) [33]. Besides insulin, Indian girls showed significantly higher C–peptide levels than boys. C–peptide, a product of insulin biosynthesis, is released in equimolar amount as mature insulin [44]. In view of higher secretion of insulin in girls, higher levels of C–peptide were anticipated [44, 45, 46]. Furthermore, mean HbA_1_c levels in Indians (5.16%) resembled Mexican–Americans (5.05%), non–Hispanic blacks (5.07%) and non–Hispanic whites (4.93%) [47]. Despite low glucose levels, we noticed unusually high HbA_1_c in girls during adolescence. Plasma glucose molecules attach non–enzymatically to hemoglobin in erythrocytes (HbA_1_c) [48]. Any variation in hemoglobin levels due to erythrocyte turnover will influence HbA_1_c values regardless of glucose levels. During adolescence, there is an upsurge in release of sex–specific hormones–estrogen in girls and testosterone in boys. Higher testosterone synthesis in boys at puberty results in greater production of hemoglobin [49]. HbA_1_c is the ratio of glycated hemoglobin to total hemoglobin content. Hence, a higher amount of total hemoglobin in boys will end up in lower HbA_1_c levels than girls which is what we also observed in our study.

Levels of total cholesterol in Indian adolescents were found to lie between 2.49–5.54mmol/L that is correspondent to well–established levels (2.9–5.8mmol/L) in children and adolescents from multiple ethnicities in Canada [33]. We observed mean levels of TC as 3.78mmol/L that is marginally lower than previously reported TC levels (4.85mmol/L) in Indian adolescents [38]. Further, presence of high TC levels in girls than boys during adolescence is also detected in Croatians where girls display normal TC levels within 3.3–6.2mmol/L as against 2.8–5.6mmol/L in boys [36]. Identification of high TC in girls is also noticed in Chinese adolescents [50]. In each gender, TC levels remained constant across different ages during adolescence. This finding is however inconsistent with the results in US adolescents that presented a decrease in TC levels with sexual maturation [51]. It is well–established that during puberty plasma lipid levels tend to differ by gender, ethnicity or both [51]. Correspondingly normal LDL levels in Indian adolescents were found to be similar between genders ranging from 1.16–3.69mmol/L. Similarity in LDL levels between girls and boys is also seen in Europeans ({Boys: 1.5–4.1mmol/L}; {Girls: 1.6–4.3mmol/L }) [36] and previously studied Indians ({Boys: mean levels = 2.20mmol/L}; {Girls: mean levels = 2.22mmol/L}) [38]. After the age of 13 years (peak pubertal period), girls showed higher LDL levels than boys. This nicely agrees with the observations in 14-year-old Chinese adolescents (girls: 1.03–3.41mmol/L; boys: 0.97–2.99mmol/L) [50]. Puberty is accompanied by increased BMI and subcutaneous adiposity owing to abrupt rise in estrogen levels [52]. Subcutaneous adiposity is a major covariate for LDL levels in adolescents (p < 0.001) [53] which fairly accounts for raised LDL levels in girls following puberty.

Normative range of HDL levels in Indians (0.78–1.85mmol/L) is lower than previously reported limits in Europeans (0.9–2.2mmol/L) [36]. In addition, mean HDL levels were observed to be like earlier stated levels in Indian adolescents (1.22mmol/L against 1.14mmol/L) [38]. During early years of adolescence, both boys and girls harbored similar HDL levels. However, at puberty, boys uniquely encountered a radical decline in HDL levels. Essentially, male puberty is characterized by boost in testosterone production that stimulates hepatic lipase activity [54], thereby reducing plasma HDL levels during the period [55]. On the other hand, observed TG levels in Indian adolescents (0.33–2.24mmol/L) correspond to normative TG levels in children and adolescents in Canada (0.5–2.23mmol/L) [33], however disperse over a wider range than Europeans (0.3–1.6mmol/L) [36]. Mean TG levels were slightly lower than our previously reported TG levels in lesser number of Indian adolescents (1.04mmol/L than 1.56mmol/L) [38]. At all successive ages after onset of puberty (14 years), high TG levels prevailed in Indian boys. Also, in Chinese boys, TG levels were reported to significantly increase from 0.79mmol/L at 12 years to 1.00mmol/L at 18 years of age [50]. Elevated TG levels in boys during adolescence occurs due to testosterone mediated decrease in Post Heparin (PH) Lipoprotein Lipase [56], an enzyme that catalyzes hydrolysis of TG [57].

Serum urea, uric acid and creatinine levels in Indian adolescents were observed to commonly reside within respective limits of 3.56–11.45mmol/L; 130.01–440.15μmol/L and 22.99–74.28μmol/L that are wider in comparison to 2.7–6.8mmol/L, 125–228μmol/L and 46–80μmol/L in Europeans [36]. All three studied nitrogen metabolites revealed exceptionally high levels in boys than girls throughout adolescence. Urea is a chief nitrogenous waste product of dietary proteins [58]. Protein rich foods largely comprise fish, meat, poultry and eggs. We recruited our samples from Northern part of India that is mainly dominated by vegetarian population [21, 59]. Here, men are primary meat consuming people [21, 59] and therefore vindicates high urea levels in boys. Similarly, dietary intake of high amounts of purine rich foods–red meat, sea foods, mushrooms, etc. end up in high uric acid levels in blood plasma [60]. At odds with fundamental influence of diet on plasma levels of urea and uric acid, creatinine is a waste product synthesized by muscles at a steady rate as a part of normal daily activity [61]. Men have higher muscle mass than women throughout their life [61] which corresponds to high creatinine production and elevated levels in blood.

Besides drastic variation in blood biochemistry due to multiple physiological events, a higher BMI further reinforces this variability during adolescence. We observed that all biochemical parameters vary considerably among Obese/ overweight and lean adolescents. Urea was noted to be an exception to the prevalent trend. The underlying rationale behind consistency of serum urea levels with obesity is though unknown, however urea levels has been previously been reported to remain unaltered with weight loss [62].

As is now evident that normal blood biochemistry indeed changes with ethnicity and food habits, our study therefore established a population specific normative range of common blood biochemical parameters in Indian adolescents and investigated its variability with gender, age and BMI. The age of onset of puberty varies widely among individuals owing to their genetic and environmental factors starting from as early as 8 years to as late as 16 years. The precise interpretation of our data is confined by lack of information about the age of onset of puberty in our cohort. Our findings will benefit medical practitioners assessing pediatric and adolescent health in piloting a timely intervention for chronic conditions and also serve as a platform for future studies to design proper reference intervals for adolescents in different world populations.

## Supporting information

S1 Table Statistical power of the study. (DOCX)

S2 Table Calculated p values for comparison of various biochemical parameters between boys and girls of same age group. (DOCX)

S3 Table Calculated p values for comparison of various biochemical parameters among boys of different age. (DOCX)

S4 Table Calculated p values for comparison of various biochemical parameters among girls of different age groups. (DOCX)

S5 Table Variation of biochemical parameters with BMI in boys and girls during adolescence. (DOCX)

## References

[1] Kratz A, Ferraro M, Sluss PM, Lewandrowski KB. Case records of the Massachusetts General Hospital. Weekly clinicopathological exercises. Laboratory reference values. N Engl J Med. 2004; 351(15): 1548–1563. 10.1056/NEJMcpc049016 15470219

[2] Sebastiani P, Thyagarajan B, Sun F, Honig LS, Schupf N, Cosentino S, et al Age and sex distributions of age-related biomarker values in healthy older adults from the Long Life Family Study. J Am Geriatr Soc. 2016; 64(11): e189–e194. 10.1111/jgs.14522 27783390 PMC5118076

[3] Horn PS, Pesce AJ. Effect of ethnicity on reference intervals. Clin Chem. 2002; 48(10): 1802–1804. 12324505

[4] Lim E, Miyamura J, Chen JJ. Racial/Ethnic-Specific Reference Intervals for Common Laboratory Tests: A Comparison among Asians, Blacks, Hispanics, and White. Hawaii J Med Public Health. 2015; 74(9): 302–310. 26468426 PMC4578165

[5] Du Y, Sun H, Li Y, Shi X, Pei D, Qi D, et al Reference intervals for six lipid analytes in 8–14 year-old school children from three different ethnical groups in the HulunBuir area of China. Clin Lab. 2014; 60(8): 1295–1300. 25185414

[6] Romeo J, Wärnberg J, Gómez‐Martínez S, Díaz LE, Moreno LA, Castillo MJ, et al Haematological reference values in Spanish adolescents: the AVENA study. Eur J Haematol. 2009; 83(6): 586–594. 10.1111/j.1600-0609.2009.01326.x 19659559

[7] Dosoo DK, Kayan K, Adu-Gyasi D, Kwara E, Ocran J, Osei-Kwakye K, et al Haematological and Biochemical Reference Values for Healthy Adults in the Middle Belt of Ghana. PLoS ONE. 2012; 7(4): e36308 10.1371/journal.pone.0036308 22558429 PMC3338654

[8] Ashavaid TF, Todur SP and Dherai AJ. Establishment of reference intervals in Indian population. Indian J Clin Biochem. 2005; 20(2): 110–118. 10.1007/BF02867409 23105542 PMC3453844

[9] Adeli K, Higgins V, Nieuwesteeg M, Raizman JE, Chen Y, Wong SL, et al Biochemical marker reference values across pediatric, adult, and geriatric ages: establishment of robust pediatric and adult reference intervals on the basis of the Canadian Health Measures Survey. Clin Chem. 2015; 61(8): 1049–1062. 10.1373/clinchem.2015.240515 26044506

[10] Lopez-Otin C, Blasco MA, Partridge L, Serrano M, Kroemer G. The hallmarks of aging. Cell. 2013; 153 (6): 1194–1217. 10.1016/j.cell.2013.05.039 23746838 PMC3836174

[11] Nathan Wetherill Shock. Article—Human aging. Encyclopædia Britannica, inc. Date Published: November 23, 2017. Online at https://www.britannica.com/science/human-aging, Access Date: April 25, 2018.

[12] Carol M. Worthman and Kathy Trang. Dynamics of body time, social time and life history at adolescence. Nature. 2018; 554: 445–457. 10.1038/nature25750 29469099

[13] The Editors of Encyclopaedia Britannica. Article—Puberty. Encyclopædia Britannica, inc. Date Published: April 05, 2018. Online at https://www.britannica.com/science/puberty, Accessed: 25 April 2018.

[14] Nu CT, MacLeod P, Barthelemy J. Effects of age and gender on adolescents' food habits and preferences. Food Qual Prefer. 1996; 7(3): 251–262.

[15] Conner M. Accounting for gender, age and socioeconomic differences in food choice. Appetite. 1994; 123:195. 10.1006/appe.1994.1048 7864614

[16] Antonishak J, Sutfin EL, Reppucci ND. Community Influence on Adolescent Development In: Gullotta TP, Adams GR. (eds) Handbook of Adolescent Behavioral Problems. Springer, Boston, MA 2005; 57–78.

[17] David Wakefield W. & Cynthia Hudley. Ethnic and Racial Identity and Adolescent Well-Being, Theory Into Practice. 2009; 46(2): 147–154.

[18] Trading Economics. Online at https://tradingeconomics.com/india/population. Accessed: 29 May 2018.

[19] Majumder PP, Mukherjee BN. Genetic Diversity and Affinities among Indian Populations: An Overview In: Majumder PP. (eds) Human Population Genetics. Springer, Boston, MA 1993; 255–275.

[20] Misra A, Khurana L, Isharwal S, Bhardwaj S. South Asian diets and insulin resistance. Br J Nutr. 2009; 101(4): 465–473. 10.1017/S0007114508073649 18842159

[21] Satija A, Hu FB, Bowen L, Bharathi AV, Vaz M, Prabhakaran D, et al Dietary patterns in India and their association with obesity and central obesity. Public Health Nutr. 2015;18(16): 3031–3041. 10.1017/S1368980015000312 25697609 PMC4831640

[22] Misra A, Wasir JS, Vikram NK. Carbohydrate diets, postprandial hyperlipidaemia, abdominal obesity and Asian Indians: a recipe for atherogenic disaster. Indian J Med Res. 2005; 121(1): 5–8. 15713972

[23] Feng R, Du S, Chen Y, Zheng S, Zhang W, Na G, et al High carbohydrate intake from starchy foods is positively associated with metabolic disorders: a Cohort Study from a Chinese population. Sci Rep. 2015; 5:16919 10.1038/srep16919 26581652 PMC4652281

[24] Ha K, Kim K, Chun OK, Joung H, Song Y. Differential association of dietary carbohydrate intake with metabolic syndrome in the US and Korean adults: data from the 2007–2012 NHANES and KNHANES. Eur J Clin Nutr. 2018 10.1038/s41430-017-0031-8 29339830

[25] INdianDIabetesCOnsortium. INDICO: the development of a resource for epigenomic study of Indians undergoing socioeconomic transition. HUGO J. 2011; 5(1–4): 65–69. 10.1007/s11568-011-9157-2 23205164 PMC3238020

[26] Centers for Disease Control and Prevention. CDC Growth Charts: United States. Available at: http://www.cdc.gov/growthcharts/ Accessed: December 2018.

[27] Must A and Anderson SE. Body mass index in children and adolescents: considerations for population-based applications. Int. J. of Obesity 2006; 30: 590–594. 10.1038/sj.ijo.0803300 16570087

[28] Tabassum R, Mahendran Y, Dwivedi OP, Chauhan G, Ghosh S, Marwaha RK, et al Common variants of IL6, LEPR, and PBEF1 are associated with obesity in Indian children. Diabetes. 2012; 61(3): 626–631. 10.2337/db11-1501 22228719 PMC3282821

[29] Sullivan KM, Dean A, Soe MM. OpenEpi: A Web-based Epidemiologic and Statistical Calculator for Public Health. Public Health Rep. 2009; 124(3): 471–474. 10.1177/003335490912400320 19445426 PMC2663701

[30] Faul F, Erdfelder E, Lang AG, Buchner A. G*Power 3: a flexible statistical power analysis program for the social, behavioral, and biomedical sciences. Behav Res Methods. 2007; 39(2): 175–191. 17695343 10.3758/bf03193146

[31] Soliman A, De Sanctis V, Elalaily R. Nutrition and pubertal development. Indian J Endocrinol Metab. 2014; 18, Suppl S1: 39–47. 25538876 10.4103/2230-8210.145073 PMC4266867

[32] Spear LP. The psychobiology of adolescence In: Kline K, editor. Authoritative Communities: The Scientific Case for Nurturing the Child (The Search Institute Series on Developmentally Attentive Community and Society). Springer; New York, NY: 2007 pp. 263–280.

[33] Colantonio DA, Kyriakopoulou L, Chan MK, Daly CH, Brinc D, Venner AA, et al Closing the gaps in pediatric laboratory reference intervals: a CALIPER database of 40 biochemical markers in a healthy and multiethnic population of children. Clin Chem. 2012; 58(5): 854–868. 10.1373/clinchem.2011.177741 22371482

[34] Xu L, Li M, Yin J, Cheng H, Yu M, Zhao X, et al Change of body composition and adipokines and their relationship with insulin resistance across pubertal development in obese and nonobese Chinese children: the BCAMS study. Int J Endocrinol. 2012; 2012:389108 10.1155/2012/389108 23316228 PMC3534211

[35] Oluwayemi I, Brink S, Oyenusi E, Oduwole O, Oluwayemi M. Fasting blood glucose profile among secondary school adolescents in Ado-Ekiti, Nigeria. J NutrMetab. 2015; 2015: 4. 10.1155/2015/417859 PMC4398941 25922761

[36] Jagarinec N, Flegar-Meštrić Z, Šurina B, Vrhovski-Hebrang D, Preden-Kereković V. Pediatric reference intervals for 34 biochemical analytes in urban school children and adolescents. Clinical Chemistry and Laboratory Medicine. 1998; 36(5): 327–337. 10.1515/CCLM.1998.055 9676391

[37] Aldhoon-Hainerová I, Zamrazilová H, Dušátková L, Sedláčková B, Hlavatý P, Hill M, et al Glucose homeostasis and insulin resistance: prevalence, gender differences and predictors in adolescents. DiabetolMetabSyndr. 2014; 6(1):100 10.1186/1758-5996-6-100 25419241 PMC4240882

[38] Garg M, Tandon N, Marwaha R, Singh Y. Evaluation of surrogate markers for insulin resistance for defining metabolic syndrome in urban Indian adolescents. Indian Pediatr. 2014; 51(4): 279–284. 24825264 10.1007/s13312-014-0401-4

[39] Marwaha R, Khadgawat R, Tandon N, Kanwar R, Narang A, Sastry A, et al Reference intervals of serum lipid profile in healthy Indian school children and adolescents. Clin Biochem. 2011; 44(10): 760–766. 21620812 10.1016/j.clinbiochem.2011.05.011

[40] Wayne P. Defining, establishing, and verifying reference intervals in the clinical laboratory: approved guideline third edition CLSI document C28-A3c 3rd ed Wayne (PA): Clinical and Laboratory Standards Institute 2008.

[41] Solorzano CMB, McCartney CR. Obesity and the pubertal transition in girls and boys. Reproduction. 2010; 140(3): 399–410. 10.1530/REP-10-0119 20802107 PMC2931339

[42] Moran A, Jacobs DR, Steinberger J, Hong C-P, Prineas R, Luepker R, et al Insulin resistance during puberty: results from clamp studies in 357 children. Diabetes. 1999; 48(10): 2039–2044. 10512371 10.2337/diabetes.48.10.2039

[43] Kelsey MM, Zeitler PS. Insulin Resistance of Puberty. Curr Diab Rep. 2016; 16(7): 64 10.1007/s11892-016-0751-5 27179965

[44] Rubenstein A, Steiner D. Proinsulin. Annu Rev Med. 1971; 22(1): 1–18. 4944416 10.1146/annurev.me.22.020171.000245

[45] Steiner DF, Cunningham D, Spigelman L, Aten B. Insulin biosynthesis: evidence for a precursor. Science. 1967; 157:697–700. 4291105 10.1126/science.157.3789.697

[46] Bandesh K, Podder A. C-peptide–A Promising Biomarker Beneath the Radar of Diabetes Therapeutics. J Biomol Res Ther 2018; 7:167.

[47] Saaddine JB, Fagot-Campagna A, Rolka D, Narayan KV, Geiss L, Eberhardt M, et al Distribution of HbA1c levels for children and young adults in the US: Third National Health and Nutrition Examination Survey. Diabetes Care. 2002; 25(8): 1326–1330. 12145229 10.2337/diacare.25.8.1326

[48] Stevens VJ, Vlassara H, Abati A, Cerami A. Nonenzymatic glycosylation of hemoglobin. J Biol Chem. 1977; 252(9): 2998–3002. 856810

[49] Hero M, Wickman S, Hanhijarvi R, Siimes MA, Dunkel L. Pubertal upregulation of erythropoiesis in boys is determined primarily by androgen. J Pediatr. 2005; 146(2): 245–252. 10.1016/j.jpeds.2004.09.002 15689918

[50] Hu C, Tao F, Wan Y, Hao J, Ye D. Normal reference values for serum lipid levels in Chinese adolescents between 12 and 18 years of age. J Trop Pediatr. 2009; 56(1): 13–18. 10.1093/tropej/fmp042 19506026

[51] Eissa MA, Mihalopoulos NL, Holubkov R, Dai S, Labarthe DR. Changes in fasting lipids during puberty. J Pediatr. 2016; 170: 199–205. 10.1016/j.jpeds.2015.11.018 26706233 PMC4769904

[52] Burt Solorzano CM, McCartney CR. Obesity and the pubertal transition in girls and boys. Reproduction. 2010; 140(3), 399–410. 10.1530/REP-10-0119 20802107 PMC2931339

[53] Ali O, Cerjak D, Kent JW, James R, Blangero J, Zhang Y. Obesity, central adiposity and cardiometabolic risk factors in children and adolescents: a family-based study. PediatrObes. 2014; 9(3): e58–e62. 10.1111/j.2047-6310.2014.218.x PMC4114214 24677702

[54] Herbst KL, Amory JK, Brunzell JD, Chansky HA, Bremner WJ. Testosterone administration to men increases hepatic lipase activity and decreases HDL and LDL size in 3 wk. Am J Physiol Endocrinol Metab. 2003; 284(6): E1112–E1118. 10.1152/ajpendo.00524.2002 12736156

[55] Kirkland RT, Keenan BS, Probstfield JL, Patsch W, Lin T-L, Clayton GW, et al Decrease in plasma high-density lipoprotein cholesterol levels at puberty in boys with delayed adolescence: correlation with plasma testosterone levels. J Am Med Assoc. 1987; 257(4): 502–507. 3098992

[56] Shearer GC, Joles JA, Jones H, Walzem RL, Kaysen GA. Estrogen effects on triglyceride metabolism in analbuminemic rats. Kidney Int. 2000; 57(6): 2268–2274. 10.1046/j.1523-1755.2000.00087.x 10844597

[57] Goldberg IJ. Lipoprotein lipase and lipolysis: central roles in lipoprotein metabolism and atherogenesis. Journal of Lipid Research. 1996; 37(4): 693–707. 8732771

[58] Walser M, Bodenlos LJ. Urea metabolism in man. J Clin Invest. 1959; 38(9): 1617–1626. 13842664 10.1172/JCI103940 PMC293293

[59] Government of India, Sample Registration System Baseline Survey 2014. Online at https://data.gov.in/, accessed 26 April 2018.

[60] Zgaga L, Theodoratou E, Kyle J, Farrington SM, Agakov F, Tenesa A, Walker M, McNeill G, Wright AF, Rudan I, Dunlop MG. The association of dietary intake of purine-rich vegetables, sugar-sweetened beverages and dairy with plasma urate, in a cross-sectional study. PLoS ONE. 2012; 7(6): e38123 10.1371/journal.pone.0038123 22701608 PMC3368949

[61] Chanutin A, Kinard FW. The relationship between muscle creatinine and creatinine coefficient. Journal of Biological Chemistry. 1932; 99(1): 125–134.

[62] Faber P, Johnstone AM, Gibney ER, Elia M, Stubbs RJ, Roger PL, Milne E, Buchan W and Lobley GE. The effect of rate and extent of weight loss on urea salvage in obese male subjects. British J of Nutrition 2003; 90(1): 221–231. 10.1079/bjn2003859 12844395

