# Supplementary material for: Normative range of blood biochemical parameters in urban Indian school-going adolescents
Source: PLoS One. 2019 Mar 7;14(3):e0213255. doi: 10.1371/journal.pone.0213255 (PMC6405124; doi:10.1371/journal.pone.0213255)
Supplement: S1 Table — (DOCX) [file pone.0213255.s001.docx]

**S1Table: Statistical power of the study.**

|  | **Boys** | | | **Girls** | | | **Power (%)** |
| --- | --- | --- | --- | --- | --- | --- | --- |
|  | Mean | SD | N | Mean | SD | N |  |
| **FPG (mmol/L)** | 4.909 | 0.747 | 3087 | 4.721 | 0.732 | 3890 | 100.000 |
| **Insulin (pmol/L)** | 65.344 | 54.193 | 2480 | 71.786 | 49.631 | 3056 | 99.540 |
| **C-peptide (nmol/L)** | 1.033 | 0.825 | 2189 | 1.428 | 0.837 | 2895 | 100.000 |
| **HbA1c (%)** | 5.081 | 0.566 | 1795 | 5.238 | 0.731 | 1930 | 100.000 |
|  | | | | | | | |
| **TC (mmol/L)** | 3.740 | 0.759 | 3089 | 3.812 | 0.810 | 3892 | 96.860 |
| **LDL (mmol/L)** | 2.189 | 0.621 | 3100 | 2.217 | 0.649 | 3900 | 45.100 |
| **HDL (mmol/L)** | 1.200 | 0.263 | 3099 | 1.241 | 0.285 | 3903 | 100.000 |
| **TG (mmol/L)** | 1.037 | 0.507 | 3086 | 1.034 | 0.494 | 3896 | 3.745 |
|  | | | | | | | |
| **Urea (mmol/L)** | 7.317 | 1.997 | 2302 | 6.595 | 2.004 | 2558 | 100.000 |
| **Uric acid (umol/L)** | 300.267 | 82.472 | 2419 | 239.468 | 204.101 | 2645 | 100.000 |
| **Creatinine (umol/L)** | 46.287 | 14.185 | 2296 | 41.958 | 12.890 | 2534 | 100.000 |

Power was calculated using OpenEpi and G*Power 3 for two-sided *U* test and has been shown for comparison of parameters between overall sample groups of adolescent boys and girls. N: Number of samples, SD: Standard Deviation, FPG: fasting plasma glucose, HbA_1_c: glycosylated hemoglobin, TC: total cholesterol, LDL: low-density lipoprotein cholesterol, HDL: high density lipoprotein cholesterol, TG: triglycerides.
