# Supplementary material for: Normative range of blood biochemical parameters in urban Indian school-going adolescents
Source: PLoS One. 2019 Mar 7;14(3):e0213255. doi: 10.1371/journal.pone.0213255 (PMC6405124; doi:10.1371/journal.pone.0213255)
Supplement: S2 Table — (DOCX) [file pone.0213255.s002.docx]

**S2 Table.** Calculated p values for comparison of various biochemical parameters between boys and girls of same age group.

| **Boys vs. Girls**  **(age in years)** | **11** | **12** | **13** | **14** | **15** | **16** | **17** |
| --- | --- | --- | --- | --- | --- | --- | --- |
| **FPG (mmol/L)** | 0.0003 | < 0.0001 | < 0.0001 | 0.007 | < 0.0001 | < 0.0001 | 0.001 |
| **Insulin (pmol/l)** | 0.0009 | < 0.0001 | < 0.0001 | < 0.0001 | 0.16 | 0.80 | 0.84 |
| **C-peptide (nmol/l)** | < 0.0001 | < 0.0001 | < 0.0001 | < 0.0001 | < 0.0001 | < 0.0001 | 0.046 |
| **HbA_1_c (%)** | < 0.0001 | < 0.0001 | < 0.0001 | 0.12 | 0.02 | 0.02 | 0.78 |
| **TC (mmol/L)** | 0.70 | 0.57 | 0.11 | 0.0002 | 0.02 | 0.24 | 0.57 |
| **LDL (mmol/L)** | 0.56 | 0.53 | 0.37 | 0.08 | 0.21 | 0.52 | 0.48 |
| **HDL (mmol/L)** | 0.34 | 0.48 | 0.06 | 0.0002 | < 0.0001 | < 0.0001 | < 0.0001 |
| **TG (mmol/L)** | 0.0009 | 0.01 | 0.46 | 0.22 | 0.009 | 0.002 | 0.004 |
| **Urea (mmol/L)** | < 0.0001 | < 0.0001 | < 0.0001 | < 0.0001 | < 0.0001 | < 0.0001 | < 0.0001 |
| **Uric acid (µmol/L)** | 0.005 | < 0.0001 | < 0.0001 | < 0.0001 | < 0.0001 | < 0.0001 | < 0.0001 |
| **Creatinine (µmol/L)** | 0.06 | 0.14 | 0.06 | < 0.0001 | < 0.0001 | < 0.0001 | < 0.0001 |

Mann Whitney *U* test was used to calculate p values.FPG: fasting plasma glucose, HbA_1_c: glycosylated hemoglobin, TC: total cholesterol, LDL: low-density lipoprotein cholesterol, HDL: high density lipoprotein cholesterol, TG: triglycerides.
