# Supplementary material for: Normative range of blood biochemical parameters in urban Indian school-going adolescents
Source: PLoS One. 2019 Mar 7;14(3):e0213255. doi: 10.1371/journal.pone.0213255 (PMC6405124; doi:10.1371/journal.pone.0213255)
Supplement: S3 Table — (DOCX) [file pone.0213255.s003.docx]

**S3 Table.** Calculated p values for comparison of various biochemical parameters among boys of different age.

| **Boys**  **(age in years)** | **11 vs. 12** | **12 vs. 13** | **13 vs. 14** | **14 vs. 15** | **15 vs. 16** | **16 vs. 17** |
| --- | --- | --- | --- | --- | --- | --- |
| **FPG (mmol/L)** | 0.11 | 0.20 | 0.02 | 0.17 | 0.88 | 0.28 |
| **Insulin (pmol/l)** | 0.43 | 0.74 | 0.21 | 0.38 | 0.36 | 0.15 |
| **C-peptide (nmol/l)** | 0.0005 | 0.16 | 0.70 | 0.27 | 0.79 | 0.002 |
| **HbA_1_c (%)** | 0.02 | 0.34 | 0.95 | 0.49 | 0.56 | 0.54 |
| **TC (mmol/L)** | 0.26 | 0.33 | 0.03 | 0.13 | 0.10 | 0.76 |
| **LDL (mmol/L)** | 0.24 | 0.47 | 0.62 | 0.53 | 0.16 | 0.87 |
| **HDL (mmol/L)** | 0.82 | 0.05 | 0.25 | 0.50 | 0.72 | 0.96 |
| **TG (mmol/L)** | 0.48 | 0.50 | 0.90 | 0.04 | 0.33 | 0.55 |
| **Urea (mmol/L)** | 0.04 | 0.99 | 0.40 | 0.46 | 0.006 | 0.90 |
| **Uric acid (µmol/L)** | 0.0002 | < 0.0001 | < 0.0001 | < 0.0001 | 0.42 | 0.63 |
| **Creatinine (µmol/L)** | 0.02 | 0.01 | < 0.0001 | < 0.0001 | 0.0007 | 0.20 |

Mann Whitney *U* test was used to calculate p values.FPG: fasting plasma glucose, HbA_1_c: glycosylated hemoglobin, TC: total cholesterol, LDL: low-density lipoprotein cholesterol, HDL: high density lipoprotein cholesterol, TG: triglycerides.
