# Supplementary material for: Normative range of blood biochemical parameters in urban Indian school-going adolescents
Source: PLoS One. 2019 Mar 7;14(3):e0213255. doi: 10.1371/journal.pone.0213255 (PMC6405124; doi:10.1371/journal.pone.0213255)
Supplement: S4 Table — (DOCX) [file pone.0213255.s004.docx]

**S4 Table**. Calculated p values for comparison of various biochemical parameters among girls of different age groups.

| **Girls**  **(age in years)** | **11 vs. 12** | **12 vs. 13** | **13 vs. 14** | **14 vs. 15** | **15 vs. 16** | **16 vs. 17** |
| --- | --- | --- | --- | --- | --- | --- |
| **FPG (mmol/L)** | 0.47 | 0.09 | 0.58 | 0.09 | 0.74 | 0.45 |
| **Insulin (pmol/l)** | 0.31 | 0.01 | 0.17 | 0.26 | 0.93 | 0.008 |
| **C-peptide (nmol/l)** | 0.0002 | 0.66 | 0.92 | 0.68 | 0.34 | 0.27 |
| **HbA_1_c (%)** | 0.003 | 0.26 | 0.003 | 0.22 | 0.56 | 0.18 |
| **TC (mmol/L)** | 0.77 | 0.99 | 0.79 | 0.47 | 0.34 | 0.40 |
| **LDL-c (mmol/L)** | 0.20 | 0.42 | 0.78 | 0.69 | 0.29 | 0.98 |
| **HDL-c (mmol/L)** | 0.80 | 0.56 | 0.57 | 0.62 | 0.69 | 0.22 |
| **TG (mmol/L)** | 0.04 | 0.20 | 0.49 | 0.05 | 0.69 | 0.22 |
| **Urea (mmol/L)** | 0.92 | 0.05 | 0.87 | 0.94 | 0.007 | 0.41 |
| **Uric acid (µmol/L)** | 0.005 | 0.91 | 0.89 | 0.26 | 0.74 | 0.065 |
| **Creatinine (µmol/L)** | 0.006 | 0.03 | 0.03 | 0.006 | 0.10 | 0.14 |

Mann Whitney *U* test was used to calculate p values.FPG: fasting plasma glucose, HbA_1_c: glycosylated hemoglobin, TC: total cholesterol, LDL: low-density lipoprotein cholesterol, HDL: high density lipoprotein cholesterol, TG: triglycerides.
