# Supplementary material for: Normative range of blood biochemical parameters in urban Indian school-going adolescents
Source: PLoS One. 2019 Mar 7;14(3):e0213255. doi: 10.1371/journal.pone.0213255 (PMC6405124; doi:10.1371/journal.pone.0213255)
Supplement: S5 Table — (DOCX) [file pone.0213255.s005.docx]

**S5 Table**. Variation of biochemical parameters with BMI in boys and girls during adolescence.

**S5(a):** Variation of biochemical parameters with BMI in boys during adolescence

| **BOYS** | **Obese (OB)** | | | **Overweight (OW)** | | | **Normal weight (NW)** | | | **P value** | | |
| --- | --- | --- | --- | --- | --- | --- | --- | --- | --- | --- | --- | --- |
| **Parameters** | **N** | **Median** | **Mean** | **N** | **Median** | **Mean** | **N** | **Median** | **Mean** | **OB vs NW** | **OW vs NW** | **OB vs OW** |
| FPG (mmol/L) | 302 | 4.87  (3.71 – 5.99) | 4.88 | 369 | 4.82  (3.49 – 6.37) | 4.80 | 2412 | 4.90  (3.62 – 6.97) | 4.93 | 0.818 | 0.0041 | 0.0616 |
| Insulin (pmol/L) | 246 | 100.63  (11.28 – 303.84) | 120.61 | 308 | 79.52  (7.45 – 202.95) | 86.33 | 1925 | 47.30  (11.54 – 137.99) | 54.92 | < 0.0001 | < 0.0001 | < 0.0001 |
| C-peptide (nmol/L) | 211 | 1.52  (0.31 – 4.68) | 1.77 | 285 | 0.96  (0.19 – 3.42) | 1.30 | 1692 | 0.67  (0.18 – 2.39) | 0.90 | < 0.0001 | < 0.0001 | < 0.0001 |
| HbA1c (%) | 156 | 5.44  (4.13 – 6.43) | 5.46 | 227 | 5.14  (4.0 – 6.23) | 5.16 | 1408 | 5.06  (3.82 – 6.05) | 5.02 | < 0.0001 | 0.0019 | < 0.0001 |
| TC (mmol/L) | 302 | 4.02  (2.74 – 5.95) | 4.08 | 376 | 3.86  (2.54 – 5.78) | 3.95 | 2408 | 3.61  (2.42 – 5.23) | 3.66 | < 0.0001 | < 0.0001 | 0.0323 |
| LDL (mmol/L) | 305 | 2.37  (1.46 – 4.09) | 2.48 | 379 | 2.33  (1.21 – 4.05) | 2.42 | 2412 | 2.10  (1.13 – 3.37) | 2.12 | < 0.0001 | < 0.0001 | 0.233 |
| HDL (mmol/L) | 304 | 1.06  (0.71 – 1.49) | 1.08 | 378 | 1.14  (0.75 – 1.69) | 1.15 | 2413 | 1.18  (0.78 – 1.85) | 1.22 | < 0.0001 | < 0.0001 | 0.0001 |
| TG (mmol/L) | 211 | 1.04  (0.38 – 2.05) | 1.12 | 282 | 0.94  (0.38 – 1.83) | 0.99 | 2401 | 0.92  (0.36 – 1.97) | 0.98 | < 0.0001 | 0.476 | 0.0016 |
| Urea (mmol/L) | 172 | 6.86  (4.15 – 10.63) | 7.00 | 157 | 6.96  (4.46 – 10.61) | 7.10 | 1139 | 6.89  (4.39 – 10.45) | 6.99 | 0.882 | 0.514 | 0.535 |
| Uric acid (μmol/L) | 166 | 315.24  (208.7 – 469.82) | 324.72 | 265 | 318.81  (187.36 – 436.46) | 315.10 | 1929 | 289.67  (143.94 – 454.43) | 291.08 | < 0.0001 | < 0.0001 | 0.343 |
| Creatinine (μmol/L) | 170 | 42.44  (26.72 – 63.66) | 43.04 | 281 | 46.86  (28.29 – 81.35) | 48.80 | 1799 | 44.21  (25.64 – 72.5) | 45.34 | 0.0717 | 0.00028 | < 0.0001 |

BMI status of samples was determined by calculating BMI z-scores and BMI-for-age percentiles using CDC Growth standard charts in boys. Values for biochemical parameters have been presented as Median (2.5 percentile and 97.5 percentile) and Mean. OB: Obese; OW: Overweight; NW: Normal Weight (Lean); N: sample number. Mann Whitney U test was used to calculate p values.

**S5(b):** Variation of biochemical parameters with BMI in girls during adolescence

| **GIRLS** | **Obese (OB)** | | | **Overweight (OW)** | | | **Normal weight (NW)** | | | **P value** | | |
| --- | --- | --- | --- | --- | --- | --- | --- | --- | --- | --- | --- | --- |
| **Parameters** | **N** | **Median** | **Mean** | **N** | **Median** | **Mean** | **N** | **Median** | **Mean** | **OB vs NW** | **OW vs NW** | **OB vs OW** |
| FPG (mmol/L) | 343 | 4.70  (3.65 – 6.17) | 4.75 | 544 | 4.66  (3.53 – 5.82) | 4.66 | 3001 | 4.70  (3.51 – 6.35) | 4.73 | 0.842 | 0.1 | 0.186 |
| Insulin (pmol/L) | 302 | 96.74  (9.82 – 269.41) | 105.88 | 447 | 78.34  (8.28 – 247.27) | 89.00 | 2307 | 57.44  (12.61 – 157.05) | 63.99 | < 0.0001 | < 0.0001 | 0.0008 |
| C-peptide (nmol/L) | 284 | 1.79  (0.29 – 4.41) | 1.91 | 422 | 1.62  (0.29 – 3.60) | 1.71 | 2189 | 1.28  (0.24 – 2.94) | 1.31 | < 0.0001 | < 0.0001 | 0.017 |
| HbA1c (%) | 175 | 5.66  (4.59 – 6.58) | 5.64 | 306 | 5.62  (4.24 – 6.40) | 5.50 | 1447 | 5.16  (3.74 – 6.2) | 5.13 | < 0.0001 | < 0.0001 | 0.054 |
| TC (mmol/L) | 347 | 4.09  (2.82 – 7.6) | 4.30 | 549 | 3.86  (2.72 – 5.79) | 3.95 | 2994 | 3.68  (2.48 – 5.33) | 3.73 | < 0.0001 | < 0.0001 | < 0.0001 |
| LDL (mmol/L) | 349 | 2.42  (1.35 – 5.63) | 2.62 | 550 | 2.27  (1.28 – 3.95) | 2.35 | 2999 | 2.12  (1.14 – 3.45) | 2.15 | < 0.0001 | < 0.0001 | 0.0001 |
| HDL (mmol/L) | 348 | 1.09  (0.75 – 1.61) | 1.11 | 549 | 1.18  (0.73 – 1.70) | 1.18 | 3004 | 1.22  (0.79 – 1.92) | 1.27 | < 0.0001 | < 0.0001 | < 0.0001 |
| TG (mmol/L) | 266 | 1.10  (0.42 – 2.14) | 1.19 | 436 | 0.99  (0.35 – 1.96) | 1.02 | 2995 | 0.93  (0.32 – 1.99) | 0.99 | < 0.0001 | 0.061 | < 0.0001 |
| Urea (mmol/L) | 210 | 6.16  (3.61 – 10.58) | 6.44 | 225 | 6.35  (4.03 – 9.78) | 6.45 | 1251 | 6.21  (4.04 – 9.67) | 6.35 | 0.999 | 0.423 | 0.586 |
| Uric acid (μmol/L) | 215 | 268.85  (158.16 – 405.89) | 273.18 | 378 | 252.79  (150.65 – 361.64) | 254.01 | 2045 | 225.43  (117.24 – 356.82) | 228.16 | < 0.0001 | < 0.0001 | 0.0002 |
| Creatinine (μmol/L) | 207 | 40.67  (23.39 – 68.84) | 42.73 | 364 | 41.56  (22.99 – 64.48) | 42.76 | 1912 | 40.67  (22.99 – 65.43) | 41.09 | 0.0471 | 0.0048 | 0.808 |

BMI status of samples was determined by calculating BMI z-scores and BMI-for-age percentiles using CDC Growth standard charts in girls. Values for biochemical parameters have been presented as Median (2.5 percentile and 97.5 percentile) and Mean. OB: Obese; OW: Overweight; NW: Normal Weight (Lean); N: sample number. Mann Whitney U test was used to calculate p values.
